# High-frequency repetitive transcranial magnetic stimulation is associated with sustained improvement in cannabis use disorder: a one-year, two-phase, three-arm randomized study

**DOI:** 10.3389/fpsyt.2026.1757627

**Published:** 2026-03-30

**Authors:** Albert Kar Kin Chung, Sau Wan Tang, Cheuk Yin Tse, Y. Doug Dong, Johnson Kai Chun Law, Welton Leung

**Affiliations:** Department of Psychiatry, Li Ka Shing Faculty of Medicine, The University of Hong Kong, Hong Kong, Hong Kong SAR, China

**Keywords:** cannabis, cannabis use disorder, dependence, rTMS, treatment effect

## Abstract

**Aim:**

High-frequency repetitive transcranial magnetic stimulation (rTMS) targeting the dorsolateral prefrontal cortex (DLPFC) shows promise in treating substance use disorders, but its efficacy for cannabis use disorder (CUD) is less well established. This 12-month, two-phase, three-arm, prospective, open-label study evaluated the short- and long- term effects of high-frequency rTMS over the left DLPFC in individuals with moderate to severe CUD across three treatment schedules.

**Methods:**

In the “active rTMS phase”, 18 participants (12 men, mean age 24.89) were first randomized in a 1:1:1 ratio to receive 20 rTMS sessions with 2400 pulses of 15 Hz per session over three treatment schedules for 2, 4, or 5 weeks. All participants then entered a 12-month observational “maintenance phase” without further active rTMS treatments. Outcome assessments included Severity of Dependence Scale (SDS) score, MCQ-SF, CUD severity, frequency of use, abstinence, and CPQ at baseline, post-treatment, and at 3, 6, and 12 months.

**Results:**

All rTMS sessions were well tolerated with no significant adverse events. Across all groups, rTMS was associated with improved psychological dependence and reduced craving, DSM-5 defined CUD severity, use frequency, and related problems over 12 months (all *ps* < 0.05, hedge’s g ranged -1.86 – -0.76). No consistent differences were observed between the three schedules.

**Conclusions:**

These findings provide support for the safety and the potential short- and long-term treatment effects associated with high-frequency rTMS over the DLPFC for reducing cannabis dependence and severity of CUD. Further large-scale studies are needed to differentiate the optimal treatment scheduling when using rTMS for CUD. This study was registered at clinicaltrials.gov (NCT05292547).

**Clinical Trial Registration:**

clinicaltrials.gov, identifier NCT05292547.

## 1 Introduction

Cannabis is not only the most widely used illicit substance globally, but it also ranked as the most commonly used drug in Asia in 2023 (1). It is estimated that one in ten cannabis users develop cannabis use disorder (CUD) and, in Asia, it is the third most prevalent substance for which individuals seek treatment (1, 2). The estimated prevalence rates for CUD among individuals using cannabis range from 2.9% to 22% worldwide (3, 4). Currently, no psychotherapy or pharmacotherapy has proven sufficient in assisting long-term abstinence or sustained recovery for CUD (5, 6). Given these limitations, recent neuromodulation approaches have targeted the prefrontal cortex, where substance dependence is hypothesized to be a result of an imbalance in hyperactive emotional processing in the ventral prefrontal cortex and hypoactive executive control in the dorsal prefrontal cortex (7). Enhancement of executive control via brain stimulation may help to reduce cue-induced craving—a core salient feature in substance use disorder (SUD)—and to minimize relapse in drug addiction from cue exposure (8).

Repetitive transcranial magnetic stimulation (rTMS) is a safe, non-invasive brain stimulation technique approved for treatment-resistant depression and as an adjunct to medications for obsessive-compulsive disorder (9). In SUD, high-frequency rTMS with excitatory stimulation to the dorsolateral prefrontal cortex (DLPFC) strengthens the executive functions and cognitive control, leading to reduced craving and addiction (7, 10–12). These beneficial effects are found to be dose-dependent in alcohol and nicotine use disorders, with better treatment outcomes linked to higher number of pulses (7) and repeated treatment sessions (13). Encouraging long-term therapeutic effects from rTMS have also been observed in stimulant use disorders. In cocaine use disorder, rTMS attenuates craving, delays the first lapse of cocaine use, and improves mood symptoms in cocaine users (14–16). In methamphetamine use disorder, rTMS significantly ameliorates craving, withdrawal syndrome, cognitive deficits, and mood disturbances (17–21). In 2021, rTMS received European Union (EU) approval for treating psychoactive substance use disorder (PSUD) in adults, especially for those with stimulant use, to lessen craving and relapses (22). However, poor adherence to treatment for substance users in outpatient settings is common (23, 24) and clinical experiences reflect that there are relatively high drop-out rates before completing the whole EU-approved 13-week rTMS treatment course in the PSUD population.

Despite promising findings for other SUDs, the efficacy of rTMS targeting DLPFC for CUD and the optimal treatment schedule are less well-established (12, 25–27). Existing studies showed variable treatment outcomes depending on protocol duration. For instance, a shorter intensive protocol with 20 rTMS sessions over two weeks has demonstrated large intra-individual effect in reducing cannabis use and spontaneous cannabis craving (28). Extending the treatment schedule to four and five weeks with the same number of rTMS sessions yielded more consistent effects in reducing cannabis use frequency, albeit only trend-level improvements on cannabis craving (29, 30). Nevertheless, modifying the treatment schedules with lengthened duration have enhanced treatment retention for cannabis users to receive active rTMS, improving from 33.33% for the two-week treatment schedule (27) to 75.79%-100% for the four- to five- week schedules (29, 30).Overall, while these studies coherently support rTMS benefits in reducing cannabis consumption, they identify a critical gap in the optimal balance between treatment schedule, retention and adherence, and rTMS acute and sustained effects in CUD, in particular on craving and relapses.

On these bases, the present study examined whether 20 sessions of rTMS delivered to the DLPFC in cannabis users with CUD were associated with reductions in psychological dependence. Psychological dependence was operationalized to include craving, emotional distress, and compulsion in substance use, all of which are implicated in addiction relapses (31). We also further investigated the correlations between rTMS treatment and changes in cannabis craving, CUD severity, cannabis consumption, and prolongation of relapse-free periods over one year. Rather than using a control-group design, this study explored the short- and long- term effects associated with three different accelerated rTMS treatment schedules, ranging from two to five weeks and each involving an equal number of 20 rTMS sessions, as previously examined in the literature.

## 2 Materials and methods

### 2.1 Study design

**Figure 1:**
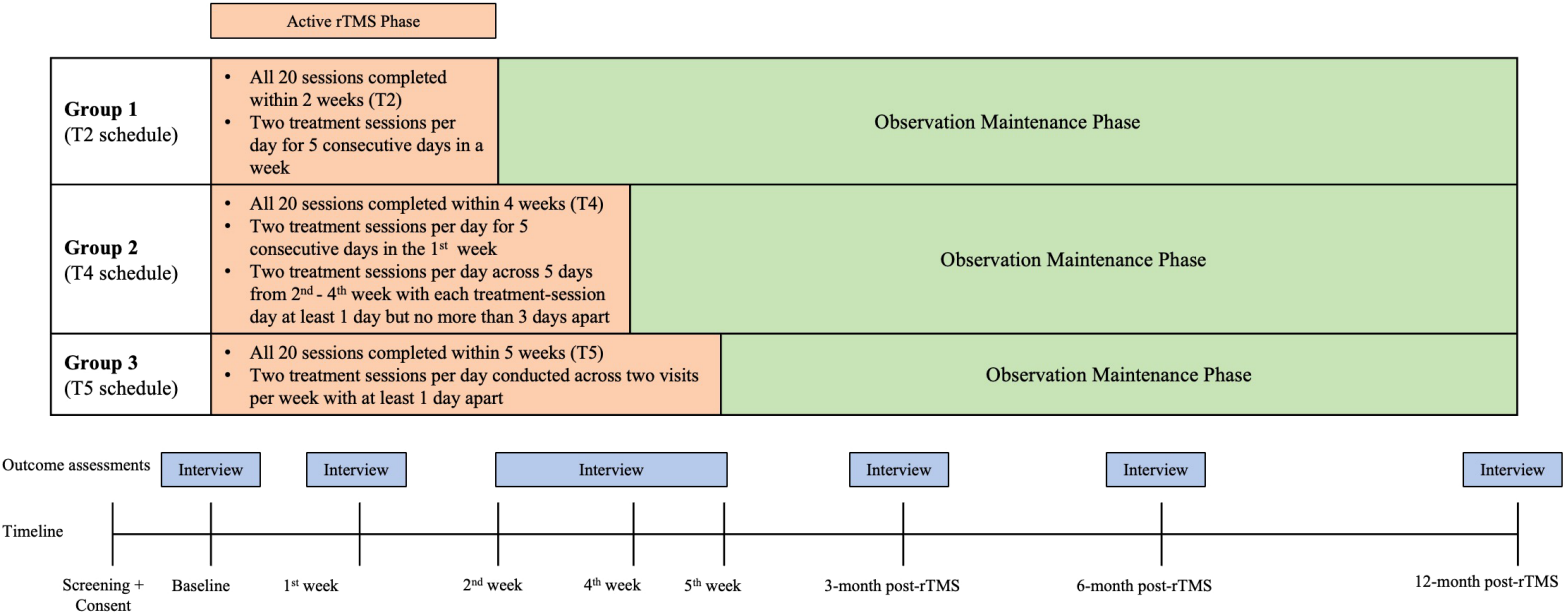
Schedules of rTMS sessions and follow-up assessment time points for the three treatment groups over the 12-month study period.

This study employed a two-phase, three-arm, prospective, open-label design over a 12-month period (**Figure 1**). In the “active rTMS phase”, participants underwent 20 rTMS treatment sessions after being randomized to three schedules: Group 1 (T2) completed 20 rTMS sessions in two weeks, with two sessions per day for five consecutive days a week; Group 2 (T4) completed two sessions per day for five consecutive days in the first week, and the rest was spread across the second to fourth week, where each treatment day was 1–3 days apart; and Group 3 (T5) completed all sessions in five weeks with two sessions per day for two non-consecutive treatment days per week. Each rTMS session per treatment-day was separated by at least a 30-minute interval. Upon completion of their rTMS sessions, participants underwent outcome assessments. After the completion of their last scheduled rTMS session, participants entered the “maintenance phase” that involved only observational follow-up assessments after 3, 6, and 12 months without further rTMS interventions. This study was approved by the Institutional Review Board of the University of Hong Kong/Hospital Authority (HA) Hong Kong West Cluster (IRB Reference: UW 21-656) and was registered at clinicaltrials.gov (NCT05292547). All study procedures adhered to the Declaration of Helsinki.

### 2.2 Prevalence and sample size

Compton and colleagues (32) suggested that the prevalence rates of DSM-5-defined mild, moderate, and severe CUD in adult users were 12.4%, 3.1%, and 1.3%, respectively. In the current study’s locality, data from the Hong Kong Central Registry of Drug Abuse between 2011 and 2020 revealed that the average number of cannabis users known to authorities was 429. Thus, the estimated numbers of users having different severities of CUD were 53 (mild), 13 (moderate), and 5 (severe). Referencing feasibility studies using rTMS targeting cannabis users with at least moderate CUD (26, 28), the sample size in this study was pre-determined to have 18 participants. So, six subjects were allocated to each of the three treatment groups.

### 2.3 Participants

Participants were recruited territory-wide in Hong Kong by snowball sampling between August 2022 to December 2023. Self-referrals, referrals from public substance misuse clinics, and non-governmental organizations were all allowed. Eligible participants were aged between 18 and 65 years and able to provide written informed consent. They should also report using cannabis as their primary substance use. The primary disorder of interest was CUD classified according to the Diagnostic and Statistical Manual of Mental Disorders 5^th^ Edition (DSM-5), and formal diagnoses of CUD were ascertained with the Structured Clinical Interview for DSM-5 Disorders: Clinician Version (SCID-5-CV) (33). Participants aged under 18 years old or those unable to provide informed consent were excluded. Participants with any comorbid psychiatric diagnosis of neurodevelopmental or neurocognitive disorders classified by DSM-5 were excluded. Participants with a comorbid SUD other than cannabis were deemed ineligible if their SUD met the moderate or severe clinical severity (DSM-5 SUD severity score ≥4). To ensure safety, any of the following conditions contra-indicating to receive rTMS precluded eligibility: having electronic and/or magnetic implants; having metallic or mechanic fragments inside the body; pregnancy; current or historical neurological conditions; poorly controlled or unstable diabetes mellitus; receiving unstable dose(s) of antipsychotics, antidepressants, benzodiazepines, and/or anticonvulsants over the past 6 months. All participants provided their written informed consent prior to any study procedure. Participants received USD$32 at the end of each treatment day during the “active rTMS phase” and at each follow-up day during the observational “maintenance phase”.

### 2.4 Procedures

Consenting participants were randomly assigned into one of the three treatment groups in a 1:1:1 ratio by a computer-generated sequence. As no sham rTMS was involved, neither participants nor researchers were blinded to the allocation. Following randomization, all participants entered the “active rTMS phase” where participants received 20 rTMS treatment sessions according to three schedules. rTMS was delivered using the MagVenture TMS system (MagPro^®^ X100) equipped with a Cool-B70 Bended Butterfly Coil at a local hospital. Stimulation targeted to the left DLPFC was localized using the BeamF3 method (34). Stimulation parameters, including frequency, train structure, total pulse number, and stimulation intensity adhered to the EU-approved MagVenture device guidance for PSUD (22, 35). Hence, the stimulation was delivered at a frequency of 15 Hz and 100% of resting motor threshold. Each train consisted of 60 pulses, with a 15-second inter-train pause, for a total of 40 trains per session, amounting to 2400 pulses per session over a 13-minute stimulation period. The resting motor threshold was determined as the minimum stimulator intensity that evoked a peak-to-peak motor-evoked potential of >50 mV in five out of ten consecutive trials.

### 2.5 Outcome measures

#### 2.5.1 Primary outcome

##### 2.5.1.1 Dependence

Psychological dependence to cannabis was measured with the five-item Severity of Dependence Scale (SDS) (36). SDS assessed the sense of control, anxiety, worry, desire, and difficulty associated with stopping cannabis use. Each item was anchored on a four-point Likert scale and the total score ranged 0 to 15. A total score ≥ 3 suggests cannabis dependence or moderate-to-severe CUD in both western and Chinese populations (37, 38).

#### 2.5.2 Secondary outcomes

##### 2.5.1.2 Craving

Cannabis craving was measured with Marijuana Craving Questionnaire – Short Form (MCQ-SF) (39). This 12-item scale captured four dimensions of craving: compulsivity, emotionality, expectancy, and purposefulness. Each item was rated on a seven-point Likert scale, with the total scores ranging 12 – 84.

##### 2.5.1.3 DSM-5 severity of CUD

Severity was assessed using SCID-5-CV (33) by a board-certified psychiatrist (author AKKC). A greater number of symptoms signified a greater severity of CUD: mild (2–3 symptoms), moderate (4–5 symptoms), and severe (6–11 symptoms). The SCID-5-CV has been translated to Chinese and certified by the American Psychiatric Association (40).

##### 2.5.1.4 Frequency

Participants reported their estimated frequency of cannabis use using the standardized Beat Drugs Fund Drug Use Frequency Questionnaire (41) available in both Chinese and English versions, which asks “In the past 3 months, how many times have you used cannabis/marijuana/grass?”. The frequency variable was manually transformed into approximate times per month, with a maximum of 30 times per month.

##### 2.5.1.5 Abstinence

Participants reported their day of the last use and the day before last with the help of a calendar. The period was then manually converted into approximate days of abstinence.

##### 2.5.1.6 Urine drug test

A urine drug test was administered at baseline and at each follow-up assessment to detect tetrahydrocannabinol (THC). This was to ensure that the participants were not under the influences of substance other than cannabis at the time of performing the rTMS.

##### 2.5.1.7 Problems related to cannabis use

Cannabis use-related problems were measured by the 22-item Cannabis Problems Questionnaire (CPQ) (42). Each item was rated on a four-point Likert scale, and the total score ranged 0 – 66.

### 2.6 Statistical analyses

Demographic information was summarized as means and standard deviations for continuous variables and counts with percentages for categorical variables. Differences in group demographics at baseline were tested using t-tests and chi-square tests where appropriate. Positive urine THC results were reported as counts and percentages. Pearson’s correlation was computed to illustrate the correlative strength between urine tests and the self-reported frequency and abstinence. Linear mixed-effects models (LMM) were employed to investigate the possible different effects over time of the rTMS intervention on psychological dependence and the secondary outcome measures of the three different rTMS treatment schedules. Fixed effects were specified for time, treatment, and their interaction, with adjustment for baseline outcome. Random intercepts were specified at participant level, but random slopes were not specified. To test the treatment effects for all participants, the time variable was modelled as a categorical variable (i.e., “post-treatment”, “3-month”, “6-month”, and “12-month”) with the grand mean at “pre-treatment” baseline serving as reference. “Post-treatment” referred to the finish timepoint of all rTMS sessions, which corresponded to the end of two weeks for T2, four weeks for T4, and five weeks for T5, respectively. To examine differences in three treatment schedules, the treatment variable was contrast-coded with T2 serving as reference: the first contrast tested whether T4 outperformed T2 and the second contrast tested whether T5 outperformed T2. Baseline outcomes were mean-centered and entered as a covariate. LMMs were estimated with restricted maximum likelihood and levels of significance were obtained with Type III ANOVA using Satterthwaite approximation. Results were reported in the format of unstandardized regression coefficients as well as standardized, model-adjusted, effect size hedge’s g for the planned contrasts. No missing data were imputed because LMM provided unbiased estimates under the assumption of data missing at random. A log-transformation was applied for days of abstinence to correct for skewness. All models converged and model diagnostics were visually inspected with no significant violations of model assumptions, nor deviations from normality or heteroscedasticity. All significance tests were two-tailed with an α level of 0.05. All regressions were conducted with packages lme4 (version 1.1-35.5), lmeTest (version 3.1-3), and emmeans (version 1.10.4) for R programming language (version 4.4.1).

## 3 Results

Two participants (one from Group 1 and one from Group 2) withdrew their consent without completing all rTMS sessions and their data were excluded. Two participants were additionally recruited to reach the targeted sample size. Therefore, the final analysis included 18 participants who completed all rTMS sessions, with three balanced groups of six participants each. All participants tolerated rTMS well with no significant adverse events reported. The most common side-effects were scalp discomfort (4.10%), dizziness (2.70%), and headache (1.91%), with no additional medical attention required (**Table 1**). Participants also showed high fidelity for completing subsequent follow-up assessments (100% at post-treatment and > 83.33% at all follow-ups) with no group differences in attendance rates (**Supplementary Table 1**). None of the participants received concurrent psychosocial or pharmacological treatments for their cannabis use during the study.

**Table 1:** Side effects reported by the 18 participants who completed the 20 rTMS sessions according to randomized schedules.

Side effects reported	Number of events (%)
Angioedema/urticaria	1 (0.27)
Anxiety	4 (0.11)
Dental Pain	3 (0.83)
Distractibility	2 (0.55)
Dizziness	10 (2.70)
Headache	7 (1.91)
Hypomania	0 (0.00)
Irritability	0 (0.00)
Nausea	4 (1.10)
Numbness	5 (1.38)
Scalp discomfort	15 (4.10)
Seizure	0 (0.00)

% was calculated based on a total of 360 rTMS sessions.

### 3.1 Baseline characteristics of the participants

Demographics and history of substance use prior to randomization were summarized in **Table 2**. Most participants were male (66.67%), single (94.44%), and in their 20s (M = 24.89, SD = 7.97). Two-thirds of the participants had received a tertiary education (66.67%). Half of the participants (55.56%) never registered with psychiatric services for their cannabis use. Two participants from Group 3 were under active psychiatric services and reported receiving antidepressants prior to enrollment. Both of them had their antidepressants stopped throughout both phases during the whole study period. No participants had been admitted to detoxification facilities before. All participants confirmed cannabis as their primary substance of use. Their average duration of cannabis use was more than five years (SD = 7.14), with an average use of around 19 days per month (SD = 11.71). Most reported active use of tobacco (88.89%) and alcohol (83.33%) and they had consumed at least one illicit psychoactive substance in the past year (M = 1.17, SD = 0.38). Lysergic acid diethylamide (LSD) (50.00%) was the most common misused substance other than cannabis among participants. Most participants (72.22%) tested positive for THC on urine drug test at baseline, and the average reported abstinence period for cannabis use was <2 weeks (SD = 3.14). Positive urine THC results largely corroborated to the self-reported frequency (*r* = 0.63) and abstinence (*r* = -0.50) of cannabis use. The SDS was 6.61 (SD = 3.22) and the average number of DSM-5 CUD symptoms was 6.50 (SD = 2.26), both reaching the clinical cut-off of “severe” CUD. Overall, participants randomized to the three treatment groups were similar in terms of demographics and baseline outcome measures, except participants from Group 3 with T5 schedule who reported no past LSD use and had lower CUD severity and fewer cannabis-related problems.

**Table 2:** Demographics and drug use history of the 18 participants.

	Total (N = 18)	Group 1 (N = 6)	Group 2 (N = 6)	Group 3 (N = 6)	*p*-value
Male, Count (%)	12 (66.67%)	3 (50.00%)	4 (66.67%)	5 (83.33%)	.
Age, Mean (SD)	24.89 (7.97)	21.33 (1.37)	25.00 (6.75)	28.33 (11.79)	.
University undergraduate, Count (%)	12 (66.67%)	4 (66.67%)	6 (100.00%)	2 (33.33%)	.
Married, Count (%)	1 (5.56%)	0 (0.00%)	0 (0.00%)	1 (16.67%)	.
Forensic history, Count (%)	3 (16.67)	1 (16.67%)	1 (16.67%)	1 (16.67%)	.
Tobacco user, Count (%)	16 (88.89%)	5 (83.33%)	6 (100.00%)	5 (83.33%)	.
Alcohol user, Count (%)	15 (83.33%)	5 (83.33%)	6 (100.00%)	4 (66.67)	.
Drinking years, Mean (SD)	6.19 (4.40)	5.83 (1.60)	8.14 (5.55)	4.60 (4.10)	.
In-patient, Count (%)	3 (16.67%)	1 (16.67%)	1 (16.67%)	1 (16.67%)	.
Out-patient, Count (%)	5 (27.78%)	2 (33.33%)	1 (16.67%)	2 (33.33%)	.
Detox center admission, Count (%)	0 (0.00%)	0 (0.00%)	0 (0.00%)	0 (0.00%)	.
Cannabis use (Mean [SD])					
Age of first use	18.28 (4.03)	17.17 (2.79)	18.83 (5.27)	18.83 (4.17)	.
Days since last use	2.67 (2.25)	2.00 (1.26)	3.33 (2.94)	2.67 (2.42)	.
Duration in months	65.28 (85.73)	53.14 (38.49)	56.57 (33.74)	95.83 (143.56)	.
Frequency in the past month	18.83 (11.71)	20.33 (8.89)	17.33 (14.79)	18.33 (12.78)	.
Lifetime history of other substance use (Count [%])					
Alprazolam	2 (11.11%)	1 (16.67%)	1 (16.67%)	0 (0.00%)	.
Cocaine	3 (16.67%)	1 (16.67%)	1 (16.67%)	1 (16.67%)	.
Ketamine	3 (16.67%)	1 (16.67%)	1 (16.67%)	1 (16.67%)	.
LSD	9 (50.00%)	5 (83.33%)	4 (66.67%)	0 (0.00%)	* T2 > T5
Magic mushrooms	4 (22.22%)	1 (16.67%)	3 (50.00%)	0 (0.00%)	.
MDMA	1 (5.56%)	1 (16.67%)	0 (0.00%)	0 (0.00%)	.
Mescaline	3 (16.67%)	2 (33.33%)	1 (16.67%)	0 (0.00%)	.
Methaqualone	1 (5.56%)	0 (0.00%)	0 (0.00%)	1 (16.67%)	.
Methamphetamine	2 (11.11%)	0 (0.00%)	1 (16.67%)	1 (16.67%)	.
Methylphenidate	1 (5.56%)	0 (0.00%)	1 (16.67%)	0 (0.00%)	.
Number of other substances used (Mean [SD])					
Within a year	1.17 (0.38)	1.17 (0.41)	1.33 (0.52)	1.00 (0.00)	.
Within 3 months	0.94 (0.54)	1.17 (0.41)	0.83 (0.75)	0.83 (0.41)	.
Outcome measures at baseline (Mean [SD])					
Primary outcome					
SDS	6.61 (3.22)	5.33 (1.63)	7.67 (1.51)	6.83 (5.19)	.
Secondary outcomes					
MCQ-SF	46.33 (15.78)	37.33 (15.71)	54.50 (7.71)	47.17 (19.04)	.
DSM-5 Severity of CUD	6.50 (2.26)	6.17 (2.48)	8.33 (1.37)	5.00 (1.55)	* T4 > T5
Frequency of cannabis use (times per month)	18.83 (11.73)	20.33 (8.89)	17.33 (14.79)	18.83 (12.78)	.
Abstinence from cannabis use (days)	12.72 (22.01)	4.25 (4.49)	21.33 (29.99)	12.58 (23.49)	.
CPQ	11.17 (4.68)	11.17 (5.27)	14.17 (2.14)	8.17 (4.54)	* T4 > T5

Asterisk (*) denotes significant group differences with Bonferroni-adjusted *p* < 0.05. CPQ, Cannabis Problems Questionnaire; CUD, cannabis use disorder; DSM-5, Diagnostic and Statistical Manual of Mental Disorders 5^th^ Edition; LSD, Lysergic Acid Diethylamide; MCQ-SF, Marijuana Craving Questionnaire – Short Form; MDMA, 3, 4-methylenedioxymethamphetamine; N, number of participants; SD, standard deviation; SDS, Severity of Dependence Scale; T2, Group 1 participants with all rTMS sessions conducted in two weeks; T4, Group 2 participants with all rTMS sessions conducted in four weeks; T5, Group 3 participants with all rTMS sessions conducted in five weeks.

### 3.2 Overall treatment effects from 20 rTMS treatment sessions to all 18 participants

#### 3.2.1 Primary outcome

**Figure 2:**
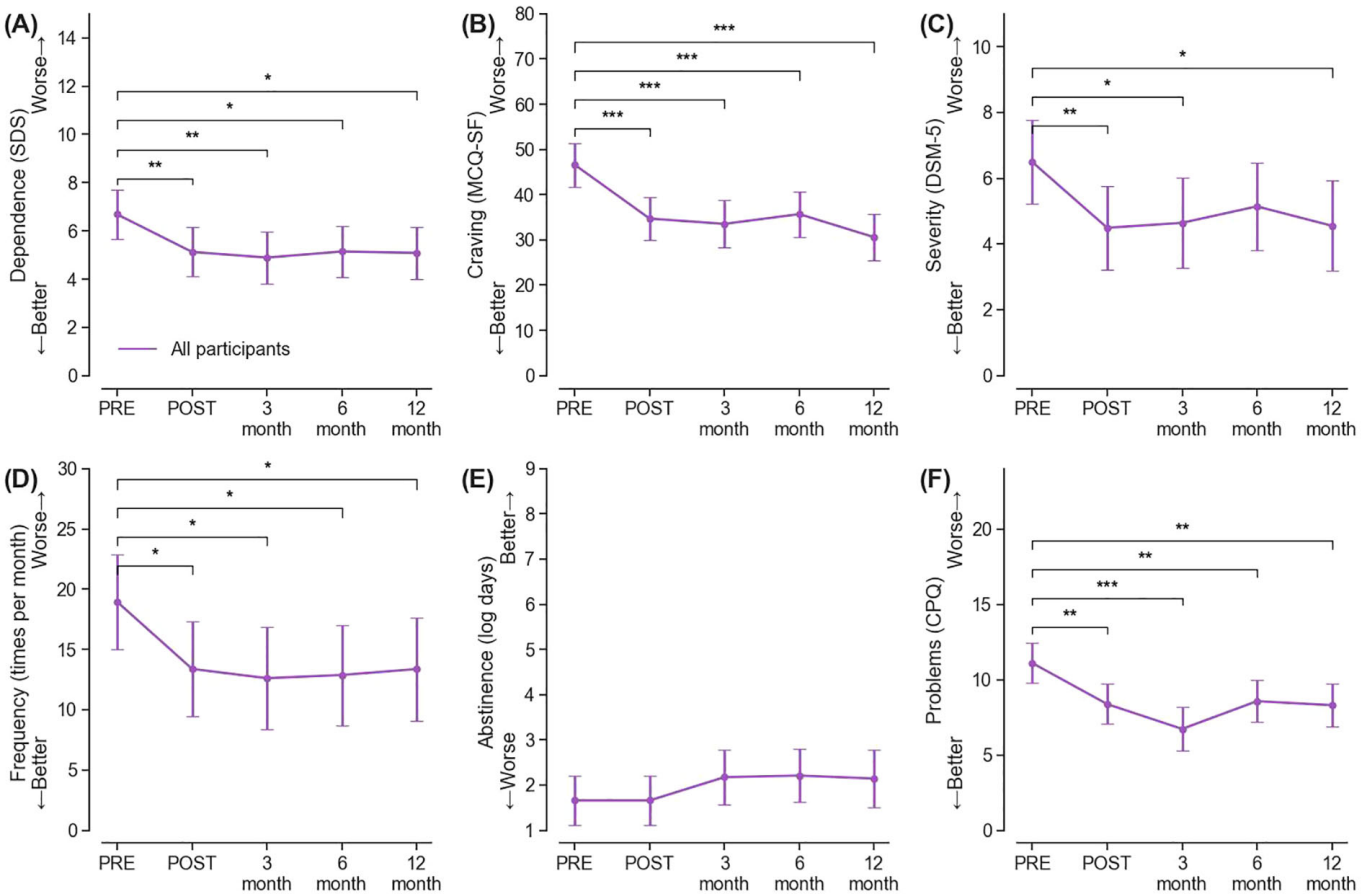
**(A–F)** represents the overall rTMS treatment effects across 12 months on Severity of Dependence (SDS), craving measured by Marijuana Craving Questionnaire – Short Form (MCQ-SF), DSM-5 defined severity of CUD, frequency of cannabis use, days of cannabis abstinence, and problems related to cannabis use measured by Problems Questionnaire (CPQ), respectively. Asterisk (*) denotes significant group differences with ***p < 0.001, **p < 0.01, and *p < 0.05.

A significant fixed effect for time was observed for psychological dependence (F(4, 54) = 3.05, *p* = 0.024), with an average reduction of 1.56 points at post-treatment (95% CI = [-2.71, -0.40], *p* = 0.009, *g* = -0.88), which was maintained at a similar magnitude at 3-month, 6-month, and 12-month follow-ups (all *ps* < 0.05, *g*s [-1.02, -0.87]) (**Figure 2A**, **Supplementary Table 2**).

#### 3.2.2 Secondary outcomes

A decrease of 11.89 points (95% CI = [-17.55, -6.23], *p* < 0.001, *g* = -1.38) in cannabis craving was observed at post-treatment, of which a benefit of similar magnitude was maintained at all subsequent follow-up timepoints (all *ps* < 0.001, gs [-1.86, -1.27]) (**Figure 2B**, **Supplementary Table 3**). The degree of CUD severity (F(4, 54) = 2.59, *p* = 0.046) also showed improvement, on average, from “severe” to “moderate” associated with rTMS treatments. Such improvement was largely maintained at subsequent follow-up timepoints (*g*s [-0.91, -0.84]), except at 6-month (**Figure 2C**, **Supplementary Table 4**). There was a trend-significant decrease in monthly frequency of cannabis use (F(4, 54) = 2.33, *p* = 0.067). Specifically, the average frequency in monthly use decreased by 5.57 times per month at post-treatment (95% CI [-10.38, -0.75], *p* = 0.024, *g* = -0.76) from 18.83 times pre-treatment, and it remained reduced at five to six fewer times per month at all follow-ups (all *ps* < 0.05, *g*s [-0.87, -0.76]) (**Figure 2D**, **Supplementary Table 5**). Nevertheless, there were no significant changes in days of abstinence during the study period (F(4, 54) = 1.01, *p* = 0.41; **Figure 2E**, **Supplementary Table 6**). A decrease of positive THC urine rates was observed over time, from 72.22% (13/18) to 50.00% at 12-month follow-up (7/14). Sustained reductions were also found in cannabis-related problems (F(4, 54) = 6.31, *p* < 0.001, *g*s [-1.69, -0.98]) (**Figure 2F**, **Supplementary Table 7**).

### 3.3 Treatment effects from three different rTMS treatment schedules

**Figure 3:**
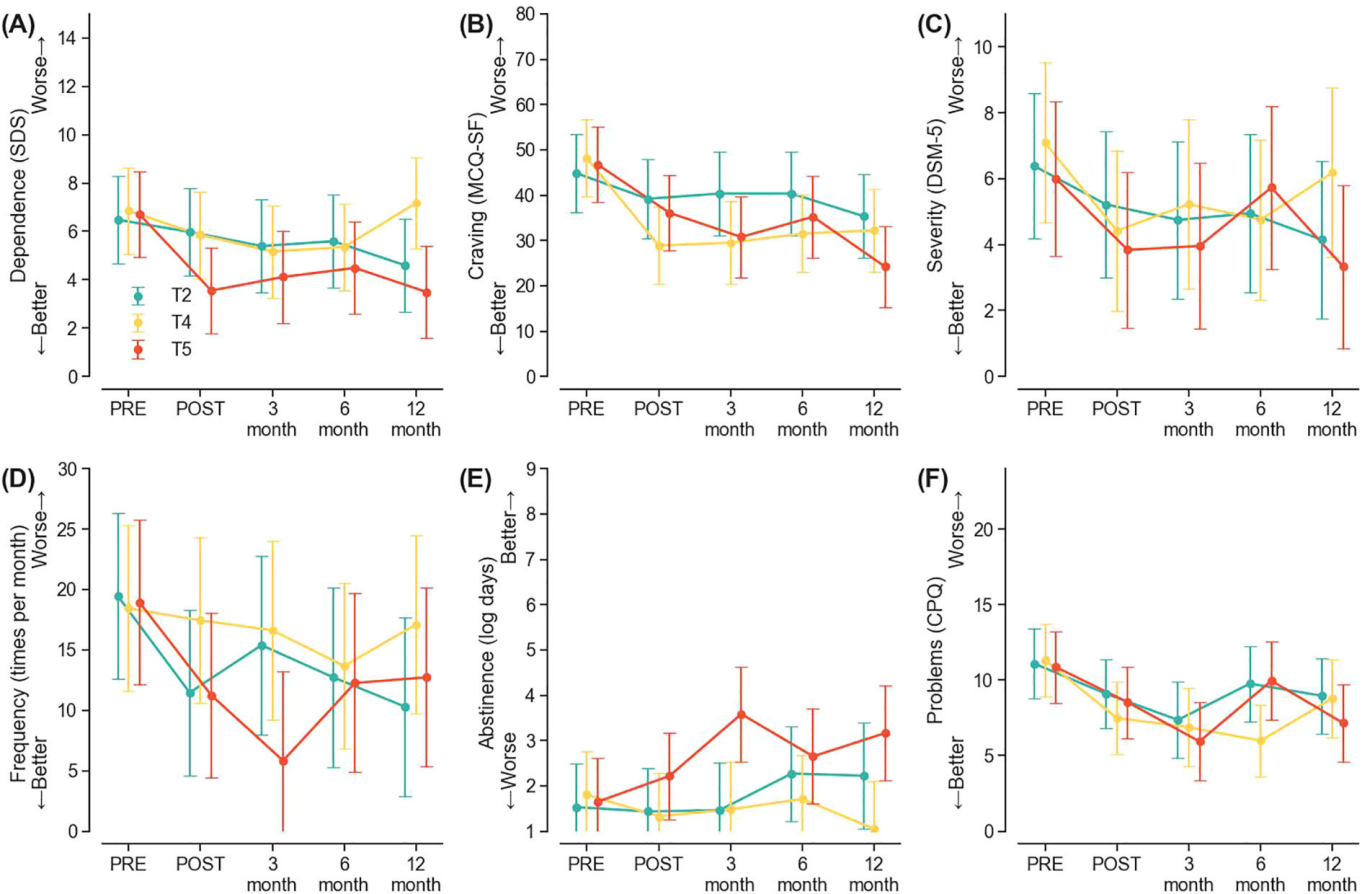
**(A–F)** represents the rTMS treatment effects of the three treatment groups across 12 months on Severity of Dependence (SDS), craving measured by Marijuana Craving Questionnaire – Short Form (MCQ-SF), DSM-5 defined severity of CUD, frequency of cannabis use, days of cannabis abstinence, and problems related to cannabis use measured by Problems Questionnaire (CPQ), respectively.

No significant treatment by time interactions were found for any of the outcome measures (all *ps* > 0.05), indicating that the three treatment schedules did not have systematically different response trajectories over the study period. (**Figures 3A–F**). Group 3 with the T5 schedule did report greater days of abstinence than Group 1 with the T2 schedule at the 3-month follow-up (B = 2.00, 95%CI = [0.03, 3.98], *p* = 0.047), though such a difference was not maintained at later points (**Figure 3E**, **Supplementary Table 6**).

## 4 Discussion

In the present study, 20 sessions of high-frequency rTMS over left DLPFC demonstrated good tolerability, and significant reductions in cannabis dependence were observed over time. Concurrent decreases in craving, severity of CUD, frequency of consumption, and related problems were also noted. These improvements were noticeable as early as post-treatment and persisted throughout the 12-month follow-up. Our results did not indicate a statistically robust advantage favoring any one schedule over the others.

The observed longitudinal reductions in cannabis dependence are broadly consistent with existing evidence of high-frequency rTMS in alcohol, nicotine, and stimulant dependence. The improvements observed in cannabis craving, cannabis consumption, and cannabis use-related problems complement current literature suggesting that excitatory rTMS enhances DLPFC-related executive control and scaffold improvements in substance use related behavioral changes (20, 26, 28, 43–45). However, days of cannabis abstinence were not significantly different from baseline after rTMS treatments. It therefore highlighted the critical need for developing a more comprehensive treatment plan involving other modalities, such as motivational interviewing and motivational enhancement therapy, to enhance the possibility of complete abstinence (46). Alternatively, it is possible that abstinence outcomes are dose-dependent and may require a higher cumulative number of rTMS sessions or maintenance stimulation to produce measurable effects as in other substance addiction (7, 12, 13, 47).

The lack of evidence favoring any of the treatment schedules is noteworthy. Although there were some benefits to the T5 schedule in extending days of abstinence at the 3-month follow-up, it diminished over time. This observation aligns with other studies suggesting that the cumulative dose of rTMS, reflected by the total number of pulses and sessions administered, might exert greater influence on clinical outcomes than the specific distribution or frequency of sessions (7, 47). Thus, these results underscore the need for further research into both optimal “dosing” and refining session scheduling from the current study within a larger sample.

Several limitations warrant considerations. The lack of power analysis in determining the sample size and the small sample size in this study came with low statistical power, which is expected to obscure meaningful group differences. The open-label, non-sham-controlled design precludes causal inference regarding rTMS-specific treatment effects, and improvements observed over time may be confounded with other factors such as expectancy effects, increased monitoring on cannabis use, or regression to the mean. Moreover, although scalp-based BeamF3 approach approximates the magnetic resonance imaging (MRI)-guided neuro-navigation for localizing the DLPFC in practice (48), using the BeamF3 method might still incur a lowered anatomical precision as compared to the MRI-guided neuro-navigation approach. Furthermore, the demographic homogeneity of the sample with primarily young males from a single region may limit the generalizability to a more diverse population. Also, both MCQ-SF and CPQ, which measured cannabis craving and use related problems, respectively, had not been validated in the local Chinese population previously. In addition, concurrent use of prescription medications or other substances were not tested during the “maintenance phase”, and their potential interactions with the rTMS effects cannot be ruled out.

Several strengths bolster the significance of this study. The current trial represents, to our knowledge, the first longitudinal study evaluating the rTMS effects over a 12-month period within an Asian cannabis-dependent population. In light of the current lack of standardized, effective treatment modalities for CUD, the sustained positive outcomes associated with rTMS throughout the study provide preliminary evidence to support further exploration and clinical adoption of high-frequency rTMS as an adjunctive treatment for CUD.

To conclude, the current study provides preliminary longitudinal data demonstrating that improvement in cannabis dependence was observed after 20-session high-frequency rTMS targeting the left DLPFC with accelerated treatment schedules from two to five weeks. Reduced craving, CUD severity, cannabis consumption, and related problems were also observed. Nonetheless, complete abstinence remained rare. Future research should evaluate whether combining high-frequency rTMS with structured psychotherapeutic interventions or optimizing stimulation dose and treatment schedules can further enhance short-term clinical gains and improve longer-term longitudinal treatment effects.

## Data Availability

The raw data supporting the conclusions of this article will be made available by the authors, without undue reservation.

## References

[1] United Nations Office on Drugs and Crime . World Drug Report 2025. Key Findings (2025). Available online at: https://www.unodc.org/documents/data-and-analysis/WDR_2025/WDR25_B1_Key_findings.pdf (Accessed February 1, 2026).

[2] United Nations Office on Drugs and Crime . World Drug Report 2025. Special Points of Interest (2025). Available online at: https://www.unodc.org/documents/data-and-analysis/WDR_2025/WDR25_Special_points_of_interest.pdf (Accessed February 1, 2026).

[3] Balodis I MacKillop J . Cannabis Use Disorder. In: Recent Advances in Cannabinoid Research. London: IntechOpen. (2019). doi: 10.5772/intechopen.80344, PMID:

[4] Leung J Chan GCK Hides L Hall WD . What is the prevalence and risk of cannabis use disorders among people who use cannabis? a systematic review and meta-analysis. Addictive Behaviors. (2020) 109:106479. doi: 10.1016/j.addbeh.2020.106479, PMID: 32485547

[5] Sherman BJ McRae-Clark AL . Treatment of cannabis use disorder: current science and future outlook. Pharmacotherapy. (2016) 36:511–35. doi: 10.1002/phar.1747, PMID: 27027272 PMC4880536

[6] Chung AKK Tse CY Yeung GKY Tang SW Chan WM Law JKC . Vortioxetine improves illness severity for cannabis users with anxiety and depressive symptoms in a 6-month randomized controlled study. J Subst Use Addict Treat. (2025) 169:209607. doi: 10.1016/j.josat.2024.209607, PMID: 39672338

[7] Zhang JJQ Fong KNK Ouyang R Siu AMH Kranz GS . Effects of repetitive transcranial magnetic stimulation (rTMS) on craving and substance consumption in patients with substance dependence: a systematic review and meta-analysis. Addiction. (2019) 114:2137–49. doi: 10.1111/add.14753, PMID: 31328353

[8] Ferguson SG Shiffman S . The relevance and treatment of cue-induced cravings in tobacco dependence. J Subst Abuse Treat. (2009) 36:235–43. doi: 10.1016/j.jsat.2008.06.005, PMID: 18715743

[9] Commissioner O of the FDA. FDA . FDA permits marketing of transcranial magnetic stimulation for treatment of obsessive compulsive disorder (2024). Available online at: https://www.fda.gov/news-events/press-announcements/fda-permits-marketing-transcranial-magnetic-stimulation-treatment-obsessive-compulsive-disorder (Accessed February 1, 2026).

[10] Enokibara M Trevizol A Shiozawa P Cordeiro Q . Establishing an effective TMS protocol for craving in substance addiction: Is it possible?: TMS for Craving. Am J Addict. (2016) 25:28–30. doi: 10.1111/ajad.12309, PMID: 26692110

[11] Maiti R Mishra BR Hota D . Effect of high-frequency transcranial magnetic stimulation on craving in substance use disorder: A meta-analysis. JNP. (2017) 29:160–71. doi: 10.1176/appi.neuropsych.16040065, PMID: 27707195

[12] Mehta DD Praecht A Ward HB Sanches M Sorkhou M Tang VM . A systematic review and meta-analysis of neuromodulation therapies for substance use disorders. Neuropsychopharmacol. (2024) 49:649–80. doi: 10.1038/s41386-023-01776-0, PMID: 38086901 PMC10876556

[13] Barr MS Farzan F Wing VC George TP Fitzgerald PB Daskalakis ZJ . Repetitive transcranial magnetic stimulation and drug addiction. Int Rev Psychiatry. (2011) 23:454–66. doi: 10.3109/09540261.2011.618827, PMID: 22200135

[14] Terraneo A Leggio L Saladini M Ermani M Bonci A Gallimberti L . Transcranial magnetic stimulation of dorsolateral prefrontal cortex reduces cocaine use: A pilot study. Eur Neuropsychopharmacol. (2016) 26:37–44. doi: 10.1016/j.euroneuro.2015.11.011, PMID: 26655188 PMC9379076

[15] Antonelli M Fattore L Sestito L Di Giuda D Diana M Addolorato G . Transcranial Magnetic Stimulation: A review about its efficacy in the treatment of alcohol, tobacco and cocaine addiction. Addictive Behaviors. (2021) 114:106760. doi: 10.1016/j.addbeh.2020.106760, PMID: 33316590

[16] Soomro H O’Neill-Kerr A Neal L Griffiths C Vai RD . Transcranial magnetic stimulation for the treatment of cocaine addiction. OJD. (2020) 09:26–30. doi: 10.4236/ojd.2020.92003

[17] Su H Zhong N Gan H Wang J Han H Chen T . High frequency repetitive transcranial magnetic stimulation of the left dorsolateral prefrontal cortex for methamphetamine use disorders: A randomised clinical trial. Drug Alcohol Dependence. (2017) 175:84–91. doi: 10.1016/j.drugalcdep.2017.01.037, PMID: 28410525

[18] Wang TY Fan TT Bao YP Li XD Liang CM Wang RJ . Pattern and related factors of cognitive impairment among chronic methamphetamine users. Am J Addict. (2017) 26:145–51. doi: 10.1111/ajad.12505, PMID: 28177556

[19] Lin J Liu X Li H Yu L Shen M Lou Y . Chronic repetitive transcranial magnetic stimulation (rTMS) on sleeping quality and mood status in drug dependent male inpatients during abstinence. Sleep Med. (2019) 58:7–12. doi: 10.1016/j.sleep.2019.01.052, PMID: 31042621

[20] Chen T Su H Li R Jiang H Li X Wu Q . The exploration of optimized protocol for repetitive transcranial magnetic stimulation in the treatment of methamphetamine use disorder: A randomized sham-controlled study. EBioMedicine. (2020) 60:103027. doi: 10.1016/j.ebiom.2020.103027, PMID: 32980696 PMC7522737

[21] Mansouriyeh N Mahmoud-Aliloo M Rostami R . The Effect of High-frequency Repetitive Transcranial Magnetic Stimulation on Reducing Depression and Anxiety in Methamphetamine Users. Addict Health. (2020) 12(4):278–86. doi: 10.22122/ahj.v12i4.288, PMID: 33623647 PMC7878001

[22] MagVenture . Addiction treatment and rTMS. (2021). Available online at: https://neurolite.ch/sites/default/files/Press%20release%20CE%20approvals_0.pdf (Accessed September 3, 2025).

[23] Roncero C Rodríguez-Cintas L Barral C Fuste G Daigre C Ramos-Quiroga JA . Treatment adherence to treatment in substance users referred from Psychiatric Emergency service to outpatient treatment. Actas Esp Psiquiatr. (2012) 40(2):63–9. Available online at: https://actaspsiquiatria.es/index.php/actas/article/view/688 (Accessed March 12, 2026). 22508071

[24] Girone N Cocchi M Achilli F Grechi E Vicentini C Benatti B . Treatment adherence rates across different psychiatric disorders and settings: findings from a large patient cohort. Int Clin Psychopharmacol. (2025) 40:232–41. doi: 10.1097/YIC.0000000000000557, PMID: 38813934

[25] Gorelick DA Zangen A George MS . Transcranial magnetic stimulation in the treatment of substance addiction. Ann New York Acad Sci. (2014) 1327:79–93. doi: 10.1111/nyas.12479, PMID: 25069523 PMC4206564

[26] Sahlem GL Baker NL George MS Malcolm RJ McRae-Clark AL . Repetitive transcranial magnetic stimulation (rTMS) administration to heavy cannabis users. Am J Drug Alcohol Abuse. (2018) 44:47–55. doi: 10.1080/00952990.2017.1355920, PMID: 28806104 PMC5962012

[27] Prashad S Dedrick ES To WT Vanneste S Filbey FM . Testing the role of the posterior cingulate cortex in processing salient stimuli in cannabis users: an rTMS study. Eur J Neurosci. (2019) 50:2357–69. doi: 10.1111/ejn.14194, PMID: 30290037 PMC6767056

[28] Sahlem GL Caruso MA Short EB Fox JB Sherman BJ Manett AJ . A case series exploring the effect of twenty sessions of repetitive transcranial magnetic stimulation (rTMS) on cannabis use and craving. Brain Stimulation. (2020) 13:265–6. doi: 10.1016/j.brs.2019.09.014, PMID: 31619347 PMC7263465

[29] Bidzinski KK Lowe DJE Sanches M Sorkhou M Boileau I Kiang M . Investigating repetitive transcranial magnetic stimulation on cannabis use and cognition in people with schizophrenia. Schizophr (Heidelb). (2022) 8:2. doi: 10.1038/s41537-022-00210-6, PMID: 35210458 PMC8873399

[30] Sahlem GL Kim B Baker NL Wong BL Caruso MA Campbell LA . A preliminary randomized controlled trial of repetitive transcranial magnetic stimulation applied to the left dorsolateral prefrontal cortex in treatment seeking participants with cannabis use disorder. Drug Alcohol Dependence. (2024) 254:111035. doi: 10.1016/j.drugalcdep.2023.111035, PMID: 38043228 PMC10837319

[31] Madsen MM . Psychological dependence. (2024). Available online at: https://www.ebsco.com/research-starters/psychology/psychological-dependence (Accessed January 23, 2026).

[32] Compton WM Han B Jones CM Blanco C . Cannabis use disorders among adults in the United States during a time of increasing use of cannabis. Drug Alcohol Dependence. (2019) 204:107468. doi: 10.1016/j.drugalcdep.2019.05.008, PMID: 31586809 PMC7028308

[33] First MB Williams JBW Karg RS Spitzer RL . Structured Clinical Interview for DSM-5 Disorders, Clinician Version (SCID-5-CV). Arlington, VA: American Psychiatric Association (2016).

[34] ClinicalResearcher.org . Adaptive PEST. Available online at: https://clinicalresearcher.org/F3/ (Accessed February 1, 2026).

[35] Madeo G Terraneo A Cardullo S Gómez Pérez LJ Cellini N Sarlo M . Long-term outcome of repetitive transcranial magnetic stimulation in a large cohort of patients with cocaine-use disorder: an observational study. Front Psychiatry. (2020) 11:158. doi: 10.3389/fpsyt.2020.00158, PMID: 32180745 PMC7059304

[36] Gossop M Darke S Griffiths P Hando J Powis B Hall W . The Severity of Dependence Scale (SDS): psychometric properties of the SDS in English and Australian samples of heroin, cocaine and amphetamine users. Addiction. (1995) 90:607–14. doi: 10.1046/j.1360-0443.1995.9056072.x, PMID: 7795497

[37] Swift W Copeland J Hall W . Choosing a diagnostic cut-off for cannabis dependence. Addiction. (1998) 93:1681–92. doi: 10.1046/j.1360-0443.1998.931116816.x, PMID: 9926531

[38] Chung AKK Tse CY . Determining the diagnostic cut-off on the Chinese version of severity of dependence scale for cannabis. Front Psychiatry. (2025) 15:1495119. doi: 10.3389/fpsyt.2024.1495119, PMID: 39839131 PMC11747374

[39] Heishman SJ Evans RJ Singleton EG Levin KH Copersino ML Gorelick DA . Reliability and validity of a short form of the Marijuana Craving Questionnaire. Drug Alcohol Dependence. (2009) 102:35–40. doi: 10.1016/j.drugalcdep.2008.12.010, PMID: 19217724 PMC2694410

[40] Fei LP . Structured Clinical Interview for DSM-5 Disorders, Clinician Version (SCID-5-CV). China: Peking University Press (2021). Chinese Edition.

[41] Narcotics Division . Narcotics Division, Security Bureau - Beat Drugs Fund Evaluation Question Sets. Available online at: https://www.nd.gov.hk/en/beat_questions_2010R2.html (Accessed February 1, 2026).

[42] Copeland J Gilmour S Gates P Swift W . The Cannabis Problems Questionnaire: Factor structure, reliability, and validity. Drug Alcohol Dependence. (2005) 80:313–9. doi: 10.1016/j.drugalcdep.2005.04.009, PMID: 15916867

[43] Sahlem GL Kim B Baker NL Wong BL Caruso MA Campbell LA . A preliminary investigation of repetitive transcranial magnetic stimulation applied to the left dorsolateral prefrontal cortex in treatment seeking participants with cannabis use disorder. medRxiv. (2023), 2023.07.10.23292461. 10.1016/j.drugalcdep.2023.111035 PMC10837319 38043228

[44] Belgers M Van Eijndhoven P Markus W Schene A Schellekens A . rTMS reduces craving and alcohol use in patients with alcohol use disorder: results of a randomized, sham-controlled clinical trial. JCM. (2022) 11:951. doi: 10.3390/jcm11040951, PMID: 35207224 PMC8878126

[45] Kan RLD Padberg F Giron CG Lin TTZ Zhang BBB Brunoni AR . Effects of repetitive transcranial magnetic stimulation of the left dorsolateral prefrontal cortex on symptom domains in neuropsychiatric disorders: a systematic review and cross-diagnostic meta-analysis. Lancet Psychiatry. (2023) 10:252–9. doi: 10.1016/S2215-0366(23)00026-3, PMID: 36898403

[46] Schwenker R Dietrich CE Hirpa S Nothacker M Smedslund G Frese T . Motivational interviewing for substance use reduction. Cochrane Database Syst Rev. (2023) 12:CD008063. doi: 10.1002/14651858.CD008063.pub3, PMID: 38084817 PMC10714668

[47] Song S Zilverstand A Gui W Li HJ Zhou X . Effects of single-session versus multi-session non-invasive brain stimulation on craving and consumption in individuals with drug addiction, eating disorders or obesity: A meta-analysis. Brain Stimulation. (2019) 12:606–18. doi: 10.1016/j.brs.2018.12.975, PMID: 30612944

[48] Mir-Moghtadaei A Caballero R Fried P Fox MD Lee K Giacobbe P . Concordance between beamF3 and MRI-neuronavigated target sites for repetitive transcranial magnetic stimulation of the left dorsolateral prefrontal cortex. Brain Stimul. (2015) 8:965–73. doi: 10.1016/j.brs.2015.05.008, PMID: 26115776 PMC4833442

