# Supplementary material for: High-frequency repetitive transcranial magnetic stimulation is associated with sustained improvement in cannabis use disorder: a one-year, two-phase, three-arm randomized study
Source: Front Psychiatry. 2026 Mar 30;17:1757627. doi: 10.3389/fpsyt.2026.1757627 (PMC13070918; doi:10.3389/fpsyt.2026.1757627)
Supplement: Supplementary file 1 [file Table1.docx]

**Supplementary Materials**

**Supplementary Table S1.** Attendance rates at different follow-up time points of all the three treatment groups.

|  | **Total** (N = 18) | **Group 1** (N = 6) | **Group 2** (N = 6) | **Group 3**  (N = 6) |
| --- | --- | --- | --- | --- |
| Pre-treatment | 18 (100.00%) | 6 (100.00%) | 6 (100.00%) | 6 (100.00%) |
| Post-treatment | 18 (100.00%) | 6 (100.00%) | 6 (100.00%) | 6 (100.00%) |
| 3-Month | 15 (83.33%) | 5 (83.33%) | 5 (83.33%) | 5 (83.33%) |
| 6-Month | 16 (88.89%) | 5 (83.33%) | 6 (100.00%) | 5 (83.33%) |
| 12-Month | 15 (83.33%) | 5 (83.33%) | 5 (83.33%) | 5 (83.33%) |

**Supplementary Table S2.** Results for linear mixed-effects model for cannabis dependence (SDS).

|  | B | 95% CI | *p* |
| --- | --- | --- | --- |
| (Intercept) | 1.04 | -0.83 – 2.91 | 0.269 |
| Baseline | 0.84 | 0.60 – 1.08 | **<0.001** |
| T4 (– T2) | 0.37 | -2.17 – 2.91 | 0.773 |
| T5 (– T2) | 0.24 | -2.26 – 2.74 | 0.851 |
| Post (– Pre) | -1.56 | -2.71 – -0.40 | **0.009** |
| Month 3 (– Pre) | -1.79 | -3.02 – -0.56 | **0.005** |
| Month 6 (– Pre) | -1.54 | -2.74 – -0.33 | **0.013** |
| Month 12 (– Pre) | -1.60 | -2.82 – -0.37 | **0.011** |
| T4 × Post | -0.50 | -3.34 – 2.34 | 0.726 |
| T5 × Post | -2.67 | -5.50 – 0.17 | 0.065 |
| T4 × Month 3 | -0.59 | -3.59 – 2.41 | 0.695 |
| T5 × Month 3 | -1.52 | -4.52 – 1.49 | 0.317 |
| T4 × Month 6 | -0.61 | -3.54 – 2.31 | 0.677 |
| T5 × Month 6 | -1.34 | -4.35 – 1.67 | 0.376 |
| T4 × Month 12 | 2.21 | -0.79 – 5.21 | 0.146 |
| T5 × Month 12 | -1.34 | -4.35 – 1.67 | 0.376 |

**Supplementary Table S3.** Results for linear mixed-effects model for cannabis craving (MCQ-SF).

|  | B | 95% CI | *p* |
| --- | --- | --- | --- |
| (Intercept) | 9.02 | -2.87 – 20.91 | 0.135 |
| Baseline | 0.81 | 0.57 – 1.04 | **<0.001** |
| T4 (– T2) | 3.34 | -9.07 – 15.75 | 0.593 |
| T5 (– T2) | 1.91 | -10.05 – 13.87 | 0.750 |
| Post (– Pre) | -11.89 | -17.55 – -6.23 | **<0.001** |
| Month 3 (– Pre) | -13.06 | -19.05 – -7.08 | **<0.001** |
| Month 6 (– Pre) | -10.90 | -16.78 – -5.02 | **<0.001** |
| Month 12 (– Pre) | -16.00 | -21.99 – -10.02 | **<0.001** |
| T4 × Post | -13.67 | -27.53 – 0.19 | 0.053 |
| T5 × Post | -5.00 | -18.86 – 8.86 | 0.474 |
| T4 × Month 3 | -14.19 | -28.86 – 0.47 | 0.058 |
| T5 × Month 3 | -11.50 | -26.16 – 3.17 | 0.122 |
| T4 × Month 6 | -12.17 | -26.45 – 2.11 | 0.094 |
| T5 × Month 6 | -7.05 | -21.72 – 7.63 | 0.341 |
| T4 × Month 12 | -6.47 | -21.14 – 8.19 | 0.381 |
| T5 × Month 12 | -13.05 | -27.72 – 1.63 | 0.080 |

**Supplementary Table S4.** Results for linear mixed-effects model for severity of cannabis use disorder (DSM-5).

|  | B | 95% CI | *p* |
| --- | --- | --- | --- |
| (Intercept) | 2.11 | -1.59 – 5.81 | 0.258 |
| Baseline | 0.68 | 0.14 – 1.21 | **0.014** |
| T4 (– T2) | 0.70 | -2.59 – 4.00 | 0.671 |
| T5 (– T2) | -0.38 | -3.53 – 2.77 | 0.811 |
| Post (– Pre) | -2.00 | -3.44 – -0.56 | **0.007** |
| Month 3 (– Pre) | -1.85 | -3.38 – -0.32 | **0.019** |
| Month 6 (– Pre) | -1.35 | -2.85 – 0.15 | 0.078 |
| Month 12 (– Pre) | -1.94 | -3.47 – -0.41 | **0.014** |
| T4 × Post | -1.50 | -5.04 – 2.04 | 0.400 |
| T5 × Post | -1.00 | -4.54 – 2.54 | 0.574 |
| T4 × Month 3 | -0.23 | -3.98 – 3.52 | 0.904 |
| T5 × Month 3 | -0.41 | -4.16 – 3.33 | 0.826 |
| T4 × Month 6 | -0.90 | -4.55 – 2.75 | 0.625 |
| T5 × Month 6 | 1.16 | -2.60 – 4.92 | 0.540 |
| T4 × Month 12 | 1.34 | -2.41 – 5.09 | 0.479 |
| T5 × Month 12 | -0.44 | -4.20 – 3.32 | 0.816 |

**Supplementary Table S5.** Results for linear mixed-effects model for frequency of use (in times per month).

|  | B | 95% CI | *p* |
| --- | --- | --- | --- |
| (Intercept) | 6.41 | 0.63 – 12.18 | **0.030** |
| Baseline | 0.66 | 0.44 – 0.88 | **<0.001** |
| T4 (– T2) | -1.02 | -10.68 – 8.64 | 0.833 |
| T5 (– T2) | -0.51 | -10.15 – 9.13 | 0.916 |
| Post (– Pre) | -5.57 | -10.38 – -0.75 | **0.024** |
| Month 3 (– Pre) | -6.34 | -11.42 – -1.25 | **0.015** |
| Month 6 (– Pre) | -6.07 | -11.07 – -1.07 | **0.018** |
| Month 12 (– Pre) | -5.58 | -10.67 – -0.49 | **0.032** |
| T4 × Post | 7.00 | -4.79 – 18.79 | 0.240 |
| T5 × Post | 0.30 | -11.49 – 12.09 | 0.960 |
| T4 × Month 3 | 2.26 | -10.21 – 14.73 | 0.718 |
| T5 × Month 3 | -9.05 | -21.52 – 3.42 | 0.152 |
| T4 × Month 6 | 1.96 | -10.18 – 14.11 | 0.748 |
| T5 × Month 6 | 0.05 | -12.43 – 12.53 | 0.994 |
| T4 × Month 12 | 7.84 | -4.64 – 20.32 | 0.214 |
| T5 × Month 12 | 2.95 | -9.53 – 15.43 | 0.638 |

**Supplementary Table S6.** Results for linear mixed-effects model for abstinence (in log-transformed days).

|  | B | 95% CI | *p* |
| --- | --- | --- | --- |
| (Intercept) | 0.69 | 0.02 – 1.35 | **0.044** |
| Baseline | 0.59 | 0.37 – 0.81 | **<0.001** |
| T4 (– T2) | 0.27 | -1.09 – 1.63 | 0.691 |
| T5 (– T2) | 0.12 | -1.24 – 1.47 | 0.863 |
| Post (– Pre) | -0.00 | -0.77 – 0.77 | 0.999 |
| Month 3 (– Pre) | 0.52 | -0.29 – 1.32 | 0.207 |
| Month 6 (– Pre) | 0.55 | -0.24 – 1.34 | 0.171 |
| Month 12 (– Pre) | 0.48 | -0.34 – 1.31 | 0.246 |
| T4 × Post | -0.38 | -2.26 – 1.50 | 0.691 |
| T5 × Post | 0.67 | -1.21 – 2.55 | 0.481 |
| T4 × Month 3 | -0.26 | -2.23 – 1.72 | 0.794 |
| T5 × Month 3 | 2.00 | 0.03 – 3.98 | **0.047** |
| T4 × Month 6 | -0.83 | -2.76 – 1.10 | 0.392 |
| T5 × Month 6 | 0.26 | -1.71 – 2.24 | 0.792 |
| T4 × Month 12 | -1.45 | -3.49 – 0.60 | 0.162 |
| T5 × Month 12 | 0.82 | -1.22 – 2.87 | 0.424 |

**Supplementary Table S7.** Results for linear mixed-effects model for cannabis problem (CPQ).

|  | B | 95% CI | *p* |
| --- | --- | --- | --- |
| (Intercept) | 0.85 | -1.79 – 3.49 | 0.522 |
| Baseline | 0.92 | 0.72 – 1.13 | **<0.001** |
| T4 (– T2) | 0.23 | -3.07 – 3.53 | 0.891 |
| T5 (– T2) | -0.23 | -3.53 – 3.07 | 0.891 |
| Post (– Pre) | -2.72 | -4.42 – -1.02 | **0.002** |
| Month 3 (– Pre) | -4.37 | -6.16 – -2.57 | **<0.001** |
| Month 6 (– Pre) | -2.52 | -4.29 – -0.76 | **0.006** |
| Month 12 (– Pre) | -2.79 | -4.58 – -1.00 | **0.003** |
| T4 × Post | -1.83 | -6.00 – 2.33 | 0.382 |
| T5 × Post | -0.33 | -4.50 – 3.83 | 0.873 |
| T4 × Month 3 | -0.72 | -5.11 – 3.68 | 0.746 |
| T5 × Month 3 | -1.19 | -5.59 – 3.20 | 0.589 |
| T4 × Month 6 | -4.00 | -8.29 – 0.28 | 0.066 |
| T5 × Month 6 | 0.42 | -3.98 – 4.82 | 0.850 |
| T4 × Month 12 | -0.40 | -4.80 – 4.00 | 0.856 |
| T5 × Month 12 | -1.58 | -5.98 – 2.82 | 0.475 |
